# NR5A1 prevents centriole splitting by inhibiting centrosomal DNA-PK activation and β-catenin accumulation

**DOI:** 10.1186/s12964-014-0055-9

**Published:** 2014-11-25

**Authors:** Chia-Yih Wang, Pao-Yen Lai, Ting-Yu Chen, Bon-chu Chung

**Affiliations:** Institute of Molecular Biology, Academia Sinica, Taipei, 115 Taiwan; Department of Cell Biology and Anatomy, College of Medicine, National Cheng Kung University, Tainan, 701 Taiwan; Institute of Basic Medical Sciences, College of Medicine, National Cheng Kung University, Tainan, 701 Taiwan

**Keywords:** NR5A1, Centriole splitting, DNA-PK, GSK3β, β-catenin, Cyclin A

## Abstract

**Background:**

Adrenogonadal cell growth and differentiation are controlled by nuclear receptor NR5A1 (Ad4BP/SF-1) that regulates the expression of adrenal and gonadal genes. In addition, SF-1 also resides in the centrosome and controls centrosome homeostasis by restricting the activity of centrosomal DNA-PK and CDK2/cyclin A.

**Results:**

Here we show that SF-1 depletion resulted in centriole splitting and amplification due to aberrant activation of DNA-PK in the centrosome of mouse adrenocortical Y1 cells. In the absence of SF-1, GSK3β was aberrantly phosphorylated during G1 phase and β-catenin was accumulated in the centrosome, but not in the nucleus. DNA-PK inhibitor vanillin reversed these phenomena. SF-1 overexpression led to inhibition of centrosomal DNA-PK activation caused by SF-1 depletion. Both full-length SF-1 and truncated SF-1 devoid of its DNA-binding domain rescued the multiple centrosome phenotype caused by SF-1 depletion, indicating that the effect of SF-1 in the centrosome is not contributed by its DNA-binding domain. Furthermore, SF-1 interacted with cyclin A in the centrosome, but not in the nucleus. Depletion of SF-1 also resulted in centriole splitting, genomic instability and reduced growth of mouse testicular Leydig MA10 cells.

**Conclusion:**

Centrosomal DNA-PK signaling triggers the accumulation of β-catenin, leading to centrosome over-duplication and centriole splitting. This cascade of centrosomal events results in genomic instability and reduced cell numbers.

## Background

Steroidogenic factor 1 (SF-1, NR5A1, Ad4BP) is a tissue-specific transcription factor expressed mainly in the adrenal glands and gonads. It belongs to the nuclear receptor superfamily that binds to its cognate DNA sequence to activate the expression of its target genes [1,2]. SF-1 regulates genes important for energy metabolism, steroidogenesis and reproduction. SF-1 also maintains adrenogonadal cell growth and differentiation; *SF-1* knockout mice are sex reversed and lack adrenals and gonads [3]. Being a transcription factor, SF-1 is located in the nucleus. However, SF-1 also resides in the centrosome and its centrosomal residency is required for the maintenance of centrosome homeostasis [4].

Centrosomes consist of a pair of centrioles and the surrounding pericentriolar materials (PCM). During each cell cycle, centrosomes duplicate only once in a tightly controlled manner [5,6]. The pair of centrioles are usually configured perpendicularly, but they lose this perpendicular relationship (disengage) at late mitosis/early G1 phase. This process relieves the physical constraint of centrioles to permit their duplication. The disengaged centrioles are maintained at a distance of 2 μm or less [7]. During S phase, both centrioles serve as a platform for the growth of new centrioles [8]. The duplicated centrioles are separated and form mitotic spindle poles for proper segregation of replicated chromosomes.

The distance between two disengaged centrioles are regulated by centrosomal β-catenin [9]. Increased abundance of β-catenin in the centrosome induces centrosome separation during mitosis. Upon entering mitosis, duplicated centrosomes go to the opposite sites of the nucleus forming spindle poles. Centrosome separation requires Nek2 (NIMA-related protein kinase 2), which phosphorylates and stabilizes the β-catenin in the centrosome during mitosis. Aberrant accumulation of β-catenin in the centrosome during G1/S phase causes centriole splitting to a distance of more than 2 μm between two centrioles; it also causes centriole over-duplication [7,9]. Thus the precise control of centrosomal β-catenin is important to maintain centriole configuration and copy numbers.

In steroidogenic cells, SF-1 functions as a centrosomal guardian to maintain centrosome homeostasis. SF-1 maintains centrosome copy numbers by controlling the activity of DNA-dependent protein kinase (DNA-PK) in the centrosome [10]. Centrosomal SF-1 interacts with and sequesters Ku70/80, the subunits of DNA-PK, from the catalytic subunit of DNA-PK (DNA-PKcs) to prevent the activation of centrosomal DNA-PK. Once SF-1 is depleted, DNA-PKcs is recruited to the centrosome forming an active complex with Ku subunits to phosphorylate downstream Akt; this signaling cascade induces centriole over-duplication. The activation of DNA-PK in steroidogenic cells is not due to nuclear DNA damage response, but caused by SF-1 depletion [10].

In this study we have investigated in more detail the mechanism by which SF-1 controls centrosome homeostasis. We showed that centrosomal SF-1 also maintained centriole configuration by controlling centrosomal GSK3β and β-catenin signaling. We found that SF-1 depletion led to the activation of centrosomal DNA-PK/Akt signaling pathway which further phosphorylated GSK3β, resulting in the accumulation of β-catenin and centriole splitting.

## Results

### SF-1 maintains genomic integrity and proper cell growth

**Figure 1:**
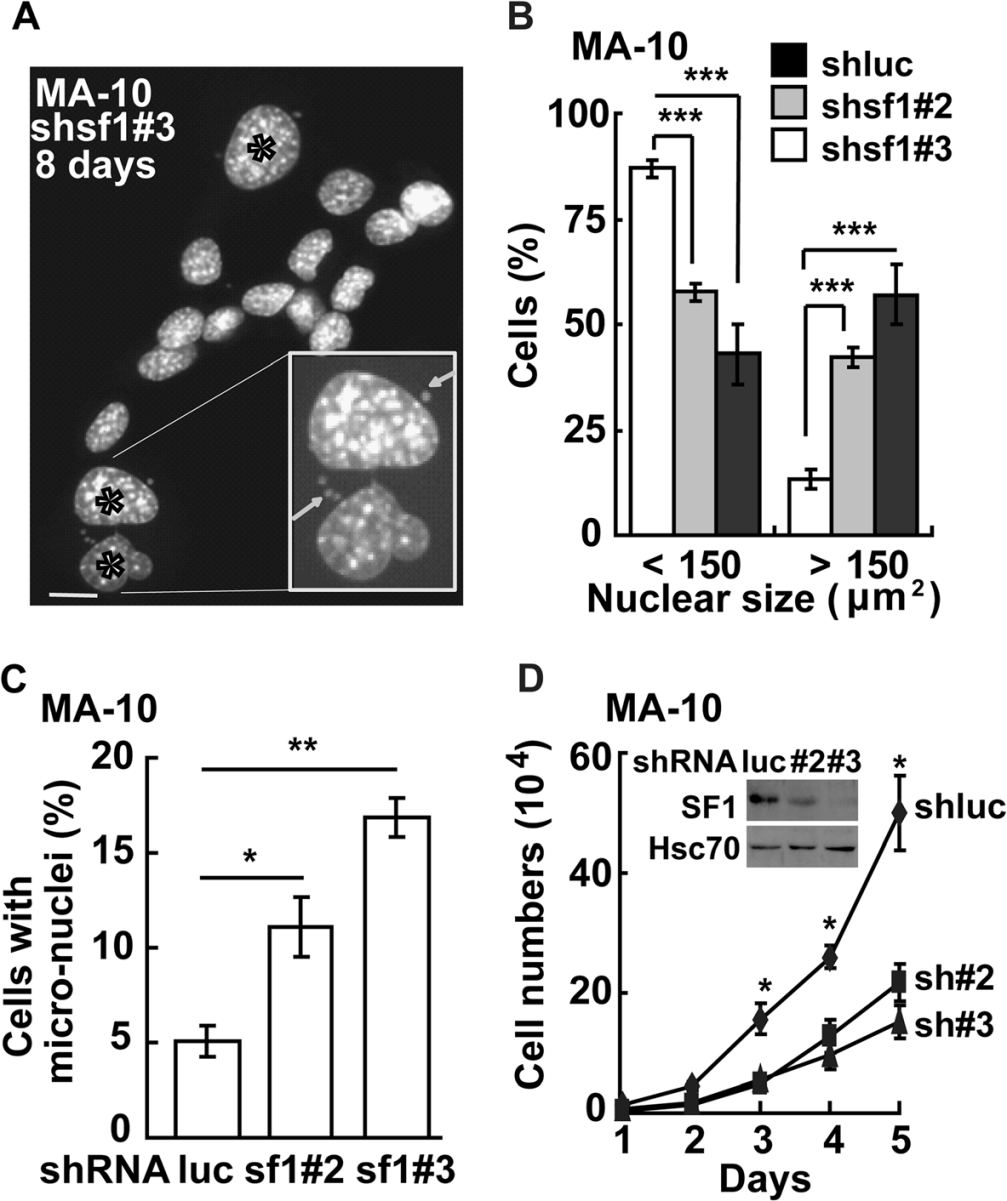
**SF-1 depletion causes genomic instability and reduced MA-10 cell growth. (A-C)** SF-1 depletion causes MA-10 genomic instability. **(A)** Staining of MA-10 nuclei with DAPI after MA-10 cells were depleted of SF-1 by the infection of *shsf1#3* lentivirus. The inset is a higher magnification showing micro-nuclei stained by DAPI. Enlarged nucleus (asterisk) and micro-nuclei (arrow) were observed. The scale bar is 5 μm. **(B-C)** Quantitation of nuclear areas **(B)** and the population of cells with micro-nuclei **(C)**. The areas of nuclei from at least 100 cells were counted and compared in three independent experiments. *P < 0.05; **P < 0.01; ***P < 0.001. At least 100 cells were counted in three independent experiments and the mean±S.D. is shown. **(D)** SF-1 depletion inhibits MA-10 cell growth. Growth curve of MA-10 cells after infection with shRNA-encoding lentivirus against luc (shluc) or two different sequences of SF-1 (sh#2, sh#3). *P < 0.05. Inset shows the Western blot analysis of SF-1 expression after infection of lentivirus for *shluc, shsf1#2 (#2) and shsf1#3 (#3)*. Hsc70 was an internal control.

SF-1 is important for genomic stability and proper growth of Y1 cells [4]. Here we tested whether the role of SF-1 can be extended to other cell types such as mouse Leydig MA-10 cells. When SF-1 was depleted by *shsf1#3* shRNA treatment for eight days, MA-10 cells contained both enlarged nuclei and micro-nuclei (Figure 1A). Counting the numbers of these nuclei, we found that most of the control *shluc* cells contained normal nuclei that were less than 150 μm^2^ in size, whereas a higher proportion of *shsf1#3* cells contained nuclei larger than 150 μm^2^ (Figure 1B). The proportions of *shsf1#3* cells with micro-nuclei that scattered around the enlarged nuclei were also increased (Figure 1C). A different shRNA sequence, *shsf1#2,* also induced the formation of bigger nuclei and micronuclei. This result indicates that MA-10 genomes were unstable when SF-1 was depleted. In addition, MA-10 cell numbers were reduced when SF-1 was depleted (Figure 1D). Thus SF-1 is important for the maintenance of genomic integrity and proper MA-10 cell growth.

**Figure 2:**
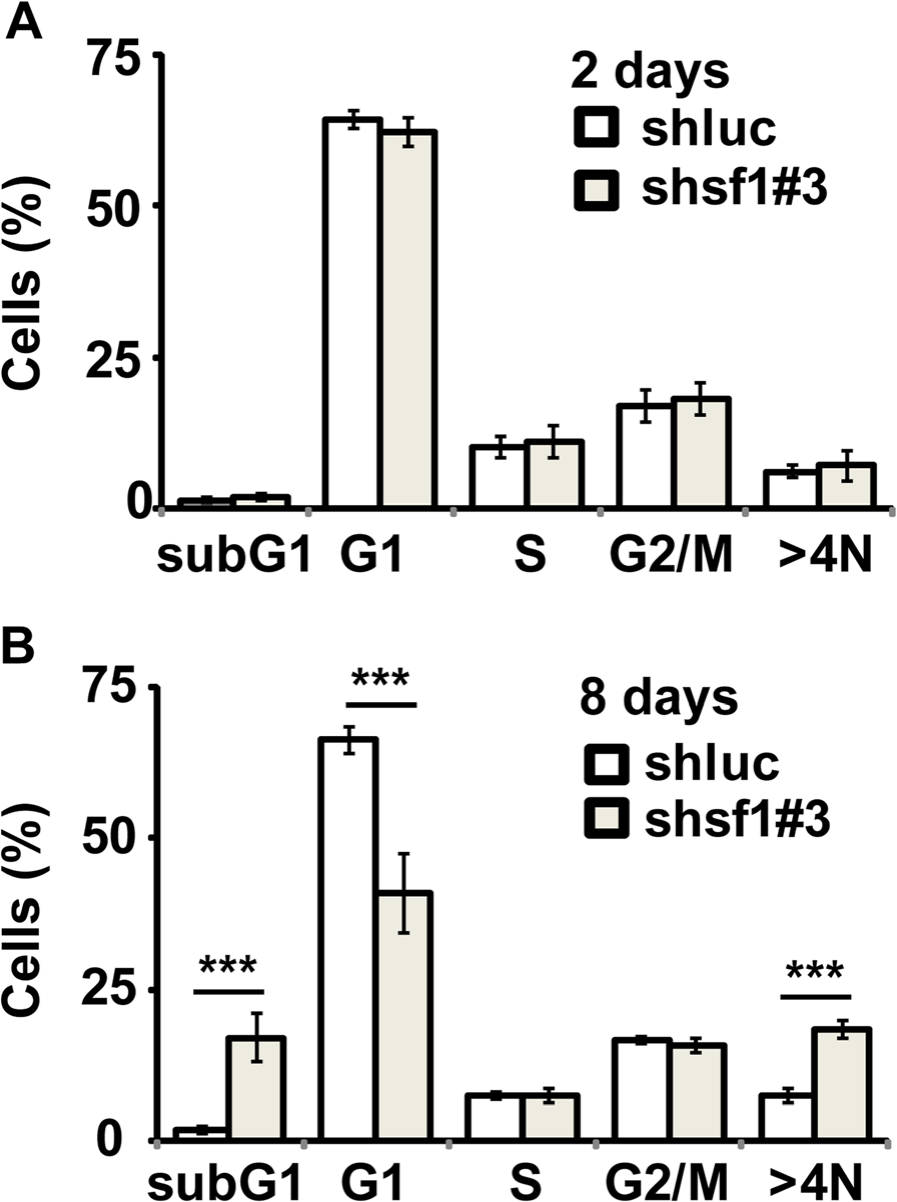
**Long-term, but not short term, SF-1 depletion disturbs cell cycle profiles.** Quantification of different cell cycle stages of Y1 cells infected with control *shluc* or *shsf1#3* lentivirus that express shRNA to deplete SF-1 for two days **(A)** or eight days **(B)** using flow cytometry.

Since SF-1 depletion affected cell growth, we examined cell-cycle profiles by flow cytometry after SF-1 depletion for 2-days or 8-days. Two days after SF-1 depletion, the proportion of cells in different cell-cycle stages was similar to that of control cells infected by *shluc* lentivirus (Figure 2A). However, when these *shsf1#3* cells were cultured for eight days, the proportions of subG1 (apoptotic cells) and polyploid cells (>4 N) were increased, whereas cells in G1 phase were reduced. These data indicated that short-term depletion of SF-1 did not disturb cell cycle progression, however, long-term depletion of SF-1 led to cell death and genomic instability.

### SF-1 restricts aberrant centriole splitting via DNA-PK

**Figure 3:**
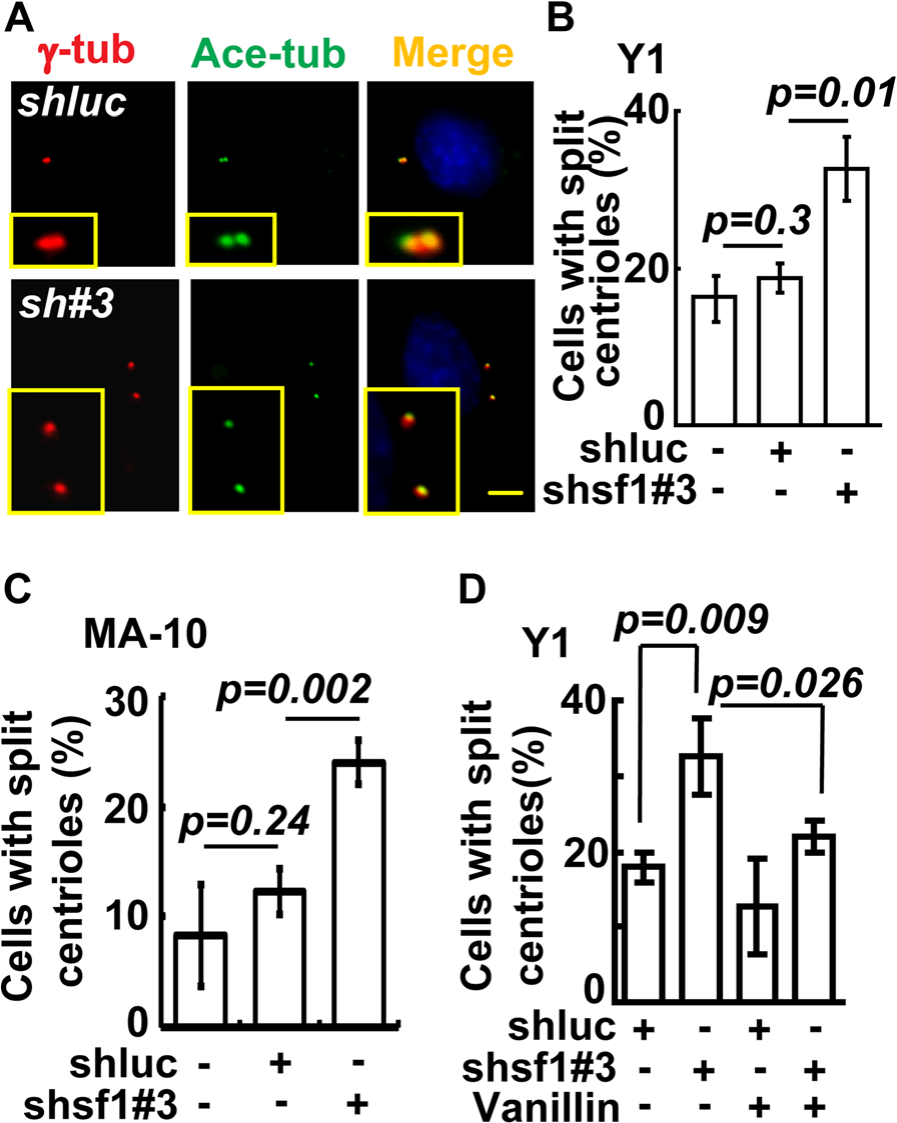
**SF-1 depletion induces centriole splitting during G1 phase. (A)** Immuno-staining of *shluc* or *shsf1#3* (sh#3) lentivirus infected Y1 cells with antibodies against γ-tubulin (γ-tub) and deploymerized acetylated α-tubulin (Ace-tub). Scale bars are 5 μm. **(B and C)** Quantification of cells with centriole distance larger than 2 μm in *shsf1#3* infected Y1 **(B)** and MA-10 **(C)** cells. **(D)** Vanillin inhibits centriole splitting induced by SF-1 depletion. Quantification of centriole splitting in *shluc* and *shsf1#3* infected Y1 cells in the presence or absence of DNA-PK inhibitor, vanillin. These results are mean ± S.D. from three independent experiments; more than three hundred cells were measured in each individual group.

We have previously shown that SF-1 depletion in Y1 cells leads to centrosome amplification [10]. The configuration of these centrioles was here examined in more detail. During G1 phase, each PCM spot stained by γ-tubulin was associated with two centrioles with a distance of less than 2 μm. However, in many *shsf1#3* cells that contained normal numbers of centrioles at G1 phase, the distance between centrioles was increased to more than 2 μm (Figure 3A), a situation defined as centriole splitting [7]. Quantitation confirmed that *shsf1#3* increased centriole splitting both in Y1 (Figure 3B) and in MA-10 cells (Figure 3C). Thus, SF-1 depletion resulted in centriole splitting.

To investigate the mechanism by which SF-1 regulates centriole splitting, we tested the involvement of DNA-PK, which triggers centrosome amplification in response to SF-1 depletion [10]. The effect of DNA-PK inhibitor vanillin on the distance between two centrioles in a cell was examined. Increased centriole splitting was observed in cells depleted of SF-1 by *shsf1#3*, but this number was reduced after vanillin treatment (Figure 3D). This result indicated that DNA-PK facilitated centriole splitting induced by SF-1 depletion in Y1 cells.

**Figure 4:**
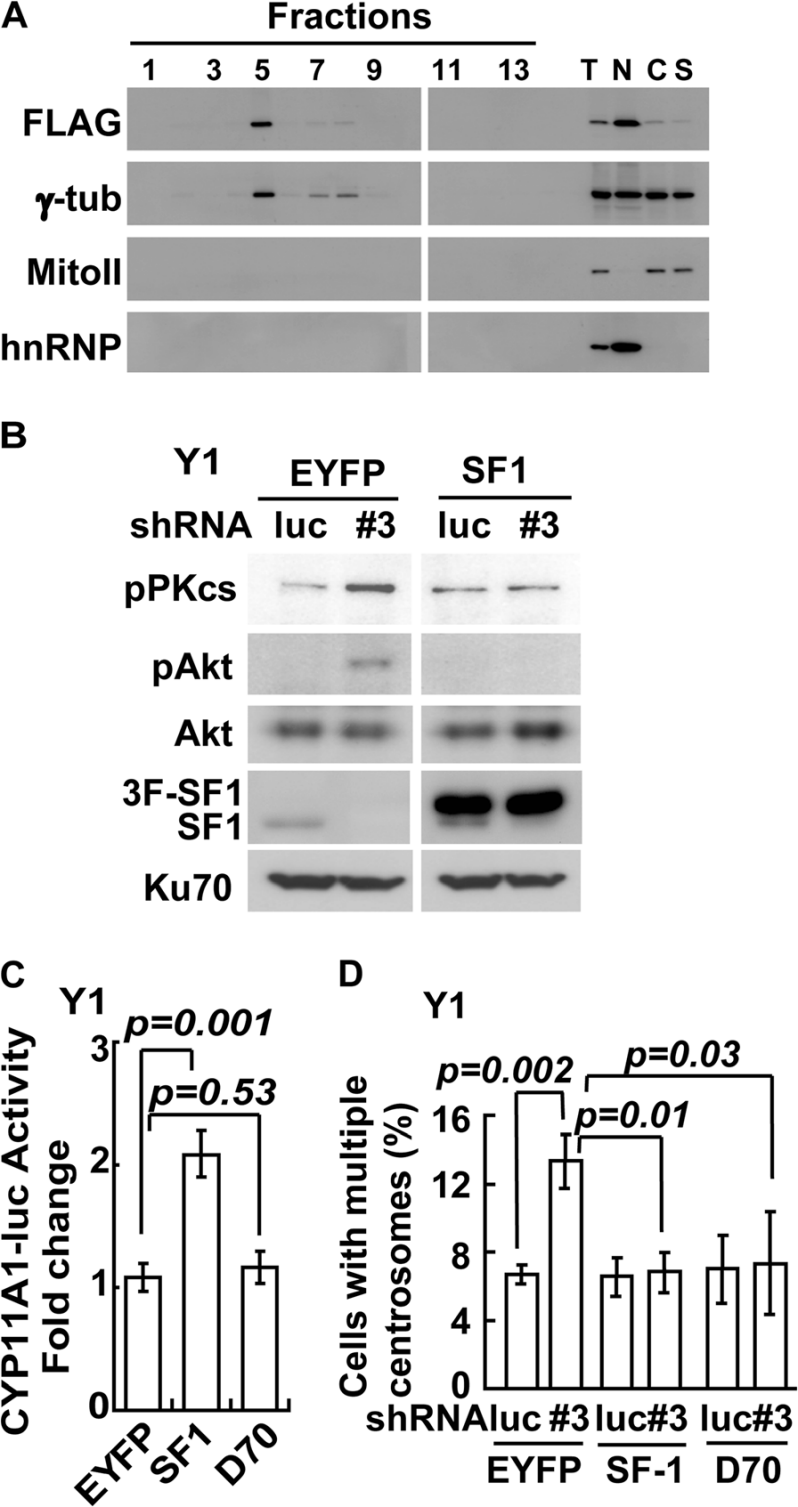
**SF-1 controls centrosome homeostasis independent of its DNA binding domain. (A)** Fractionation of centrosome from FLAG-tagged SF-1 transfected Y1 cells by sucrose gradient ultracentrifugation. Immunoblots of proteins in fractionated cell extracts are shown. Cyt: cytoplasmic fraction; Nu: nuclear fraction; Sup: post-centrosomal supernatant; WCE: whole cell extract; γ-tub: γ-tubulin; hnRNAP: hnRNP A1 (nuclear marker) and Mito II: mitochondria complex II (cytoplasmic marker). **(B)** SF-1 overexpression inhibits the activation of DNA-PK in the centrosome. Centrosomal extracts of EYFP (control) or shRNA-resistant 3-FLAG-SF-1 (3 F-SF1) overexpressing *shluc* (luc) or *shsf1#3* (#3) infected Y1 cells were analyzed by immunoblotting with antibodies against phosphorylated DNA-PKcs (pPKcs), phosphorylated Akt (pAkt), Akt, SF-1, and Ku70. For the immunoblot of SF-1, the upper band is exogenous 3 F-SF1; the lower band is endogenous SF-1 **(C)** Transcriptional activities of EYFP (negative control), wild-type SF-1 (positive control), or D70-SF-1 (D70) were measured in Y1 cells using human *CYP11A1* 2.3 k promoter linking to luciferase as a reporter. **(D)** Quantification of cells with multiple centrosomes in *shluc* (luc) or *shsf1#3* (#3) infected EYFP, SF-1 or D70 expressing Y1 cells. These results are mean ± S.D. from three independent experiments; more than three hundred cells were measured in each individual group.

To further confirm the role of SF-1 in regulating DNA-PK in the centrosome, we purified centrosomes from Y1 cells by sucrose gradient fractionation. The centrosome was enriched in fraction number 5, as shown by the presence of γ-tubulin (Figure 4A). This fraction was devoid of nuclear or cytoplasmic contaminations, as shown by the absence of mitochondrial complex II and nuclear hnRNP A1 (Figure 4A). In Y1 cells that over-expressed control EYFP, the amounts of DNA-PKcs phosphorylation was low (Figure 4B), and this amount was increased after SF-1 depletion by *shsf1#3* (*shluc*: *shsf1#3* = 1: 2.4 ± 0.5). SF-1 depletion also led to increased phosphorylation of Akt (*shluc*: *shsf1#3* = 1: 1.5 ± 0.2, Figure 4B). Overexpression of SF-1, however, blocked the phosphorylation of DNA-PKcs (*shluc*: *shsf1#3* = 1.0 ± 0. 8: 1.2 ± 0.7) and Akt (*shluc*: *shsf1#3* = 0.9 ± 0.2: 0.9 ± 0.3) in *shsf1#3* cells. Thus SF-1 prevented aberrant DNA-PK activation in the centrosome.

We also generated SF-1 that lacks the N-terminal DNA-binding domain (D70) to examine the requirement of SF-1 domains in centrosome homeostasis. The transcriptional activity of this truncated D70-SF-1 was tested after transfection with a *CYP11A1-luc* reporter gene. Wildtype SF-1, but not D70, activated reporter gene expression efficiently (Figure 4C), indicating that D70 lost transcriptional activity. We also depleted SF-1 from Y1 cells that expressed EYFP or D70 stably. A big population of EYFP-expressing cells contained multiple centrosomes when depleted of endogenous SF-1 (Figure 4D). This multiple centrosome phenotype was reversed by the re-introduction of SF-1. D70 also inhibited multiple centrosomes in SF-1-depleted Y1 cells. Thus, truncation of the DNA-binding domain abolished the transactivation function of SF-1, but did not affect its ability to maintain centrosome homeostasis.

**Figure 5:**
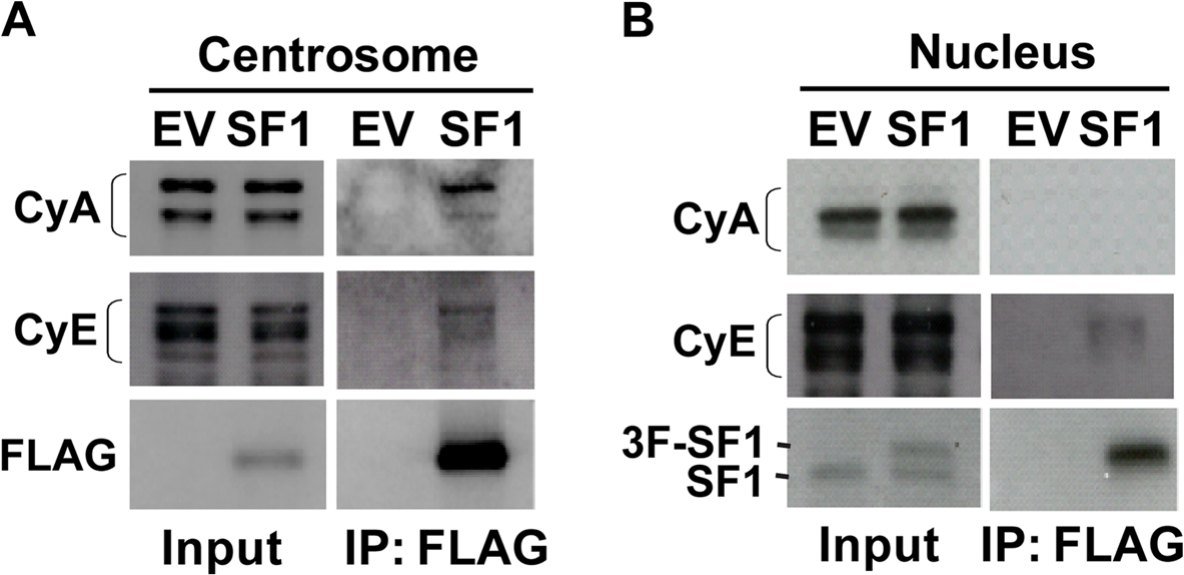
**SF-1 interacts with cyclin A and E in the centrosome. (A)** Centrosomal and **(B)** nuclear fractions of Y1 cells transfected with either empty vector (EV) or 3-FLAG-tagged SF-1 were either immunoprecipitated (IP) with anti-FLAG antibody followed by immunoblotting or directly immunoblotted with antibodies against FLAG, SF-1, cyclin A (CyA) or cyclin E (CyE).

When SF-1 is depleted, cyclin A, but not cyclin E, is accumulated in the centrosome [10]. We therefore checked whether SF-1 overexpression would affect cyclin A and cyclin E accumulation. SF-1 overexpression did not affect the amount of cyclin A and E in the centrosome, but SF-1 co-immunoprecipitated with centrosomal cyclin A (Figure 5A). In the nucleus, SF-1 did not co-immunoprecipitate with cyclin A, and only did so slightly with cyclin E (Figure 5B). The amount of cyclin A and cyclin E in the nucleus were also not affected by SF-1 overexpression. SF-1 interacted with cyclin A in the centrosome but not in the nucleus, implying that nuclear and centrosomal SF-1 proteins are associated with distinct complexes.

### Aberrant accumulation of β-catenin in the centrosome upon SF-1 depletion

**Figure 6:**
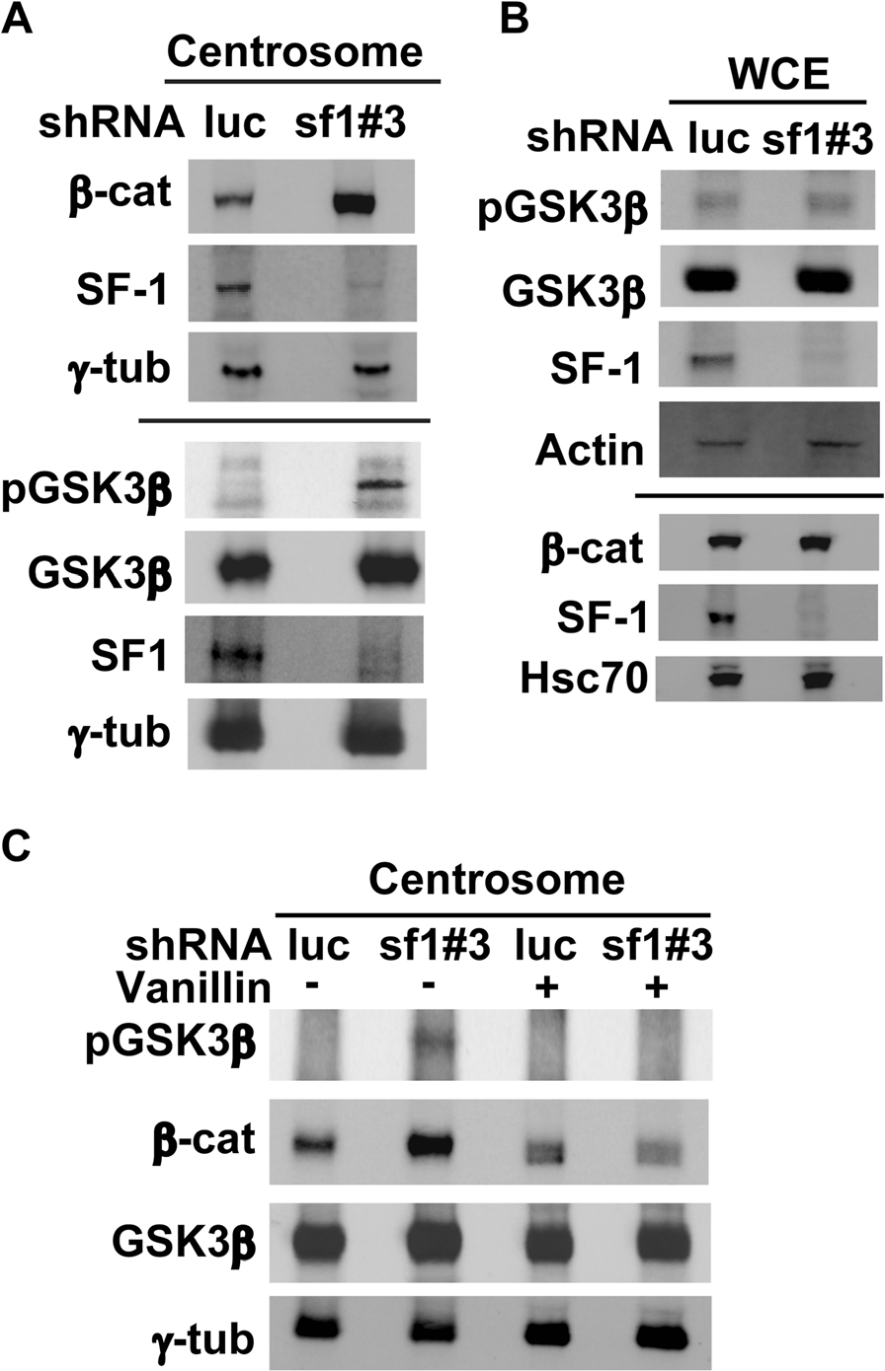
**SF-1 depletion activates DNA-PK signaling and causes accumulation of β-catenin and GSK3β in the centrosome. (A, C)** Centrosomal or **(B)** whole cell extracts (WCE) of *shluc* or *shsf1#3* lentivirus infected Y1 cells in the presence or absence of DNA-PK inhibitor, vanillin, were analyzed by immunoblotting with antibodies against β-catenin (β-cat), phosphorylated GSK3β (pGSK3β), GSK3β, SF-1, γ-tubulin (γ-tub), phsophorylated Akt (pAkt), Actin and Hsc70.

Centriole splitting depends on the accumulation of β-catenin in the centrosome at G1 phase [9]. To find out whether centriole splitting induced by SF-1 involves β-catenin, we examined the level of β-catenin after SF-1 depletion and found that it was accumulated in the centrosome (*shluc*: *shsf1#3* = 1: 2.7 ± 0.4, Figure 6A). GSK3β phosphorylation in the centrosome was also increased (*shluc*: *shsf1#3* = 1: 2.6 ± 0.4), while total amounts of GSK3β was not changed (Figure 6A). The global levels of GSK3β and its phosphorylation in the whole cell lysate, however, were not changed (Figure 6B). β-Catenin was also not accumulated in the whole cell lysate following SF-1 depletion. This result indicates that SF-1 depletion induced phosphorylation of GSK3β and accumulation of β-catenin only in the centrosome.

To investigate whether β-catenin accumulation in *shsf1#3* Y1 cells is regulated by DNA-PK, cells were treated with DNA-PK inhibitor, vanillin. Vanillin blocked phosphorylation of GSK3β (control *shluc*: control *shsf1#3*: vanillin *shluc*: vanillin *shsf1#3* = 1: 1.7 ± 0.4: 0.9 ± 0.1: 1.0 ± 0.2) as well as the accumulation of β-catenin (control *shluc*: control *shsf1#3*: vanillin *shluc*: vanillin *shsf1#3* = 1: 1.7 ± 0.2: 0.9 ± 0.2: 0.9 ± 0.3, Figure 6C). These data further indicated the importance of DNA-PK in the activation of GSK3β and the accumulation of β-catenin.

## Conclusions

In this study, we have uncovered a novel function of a tissue-specific factor SF-1 that maintains centriole configuration in adrenocortical Y1 and testicular Leydig MA-10 cells. In the centrosome, we showed that SF-1 prevented aberrant activation of the DNA-PK/Akt signaling required for centriole splitting. When SF-1 was depleted, activated DNA-PK/Akt signaling led to increased GSK3β phosphorylation followed by the accumulation of β-catenin in the centrosome, causing centriole splitting.

### SF-1 maintains centriole configuration

SF-1 is an orphan nuclear receptor that regulates the expression of genes involved in reproduction and development. In addition to being in the nucleus, SF-1 is also located in the centrosome controlling centrosome homeostasis [10]. Here we demonstrated that SF-1 depletion led to the accumulation of β-catenin followed by centriole splitting. SF-1 restrains the activity of centrosomal DNA-PK/Akt signaling by sequestering Ku from the DNA-PKcs [10]. When SF-1 is removed from the centrosome, DNA-PKcs is recruited to the centrosome and forms active complex with Ku. Following the activation of DNA-PK/Akt signaling, we showed that GSK3β was phosphorylated and inactivated leading to β-catenin accumulation in the centrosome and centriole splitting. Centriole splitting is a key step in centriole amplification in response to DNA damage [11]; here we also show that centriole splitting in SF-1-deficient Y1 also contributed to centrosome amplification.

Our previous study shows that transcriptional activity of SF-1 is dispensable for its centrosomal function [4]. In this study, we demonstrated that deletion of DNA binding domain, SF-1-D70, also prevented centrosome amplification. The DNA-binding domain of SF-1 binds DNA, however, it is still unclear whether it also has other functions in the centrosome. For example, this domain may function as a docking site of other centrosomal regulatory components. Here we showed that SF-1-D70 rescued centrosome amplification phenotype in SF-1 deficient cells, thus DNA binding domain is not involved in the regulation of centrosome homeostasis.

### SF-1 maintains adrenogonadal cell growth

SF-1 is a transcription factor that activates the transcription of adrenal and gonadal genes. In addition, it also regulates the growth of adrenogonadal cells. SF-1 appears to regulate cell growth via multiple mechanisms. Our previous publication shows that SF-1 maintains centrosome homeostasis and consequently sustains genomic stability and cell growth [4]. In the nucleus, SF-1 activates genes that promote cell proliferation [12]. In addition, it also activates genes involved in glucose metabolism as well as production of ATP and NADPH, thus maintaining proper cell growth [13]. In the absence of SF-1, cells do not produce enough energy; and cell proliferation is compromised. Thus SF-1 uses multiple methods, including the activation of genes involved in cell proliferation and energy production, as well as the assurance of centrosome homeostasis, to maintain proper cell growth. SF-1 is located both in the nucleus and the centrosome; the communication between the nucleus and the centrosome appears tight. SF-1 can function both in the nucleus and in the centrosome to maintain proper cell growth. Upon sensing a change in the environment, SF-1 can act both in the nucleus and in the centrosome to modulate cell growth. This is probably the function of a master regulator such as SF-1 in the balance of differentiation and growth of adrenogonadal cells.

### Centrosomal DNA-PK signaling regulates centrosomal β-catenin

In this report we showed that SF-1 depletion led to the induction of centriole splitting and the accumulation of β-catenin in the centrosome. β-Catenin is involved in the separation of mitotic spindle poles at the onset of mitosis [9]. During G2/M transition, β-catenin is stabilized in the centrosome to promote centrosome separation. Aberrant accumulation of β-catenin in the centrosome during G1/S causes centriole splitting and centriole over-duplication [7,9]. In our study, we found SF-1 depletion led to accumulation of β-catenin in the centrosome. This β-catenin accumulation was not due to G2/M arrest as the cell-cycle profile was not affected.

Examining the cause of β-catenin accumulation in SF-1-deficient cells, here we uncovered that GSK3β was phosphorylated by DNA-PK/Akt signaling. Phosphorylated GSK3β resulted in stabilization of β-catenin [14], and β-catenin accumulation was blocked by DNA-PK inhibitor vanillin. Thus the accumulation of β-catenin in the centrosome was due to activated DNA-PK signaling. DNA-PK is not the only DNA damage regulator that regulates β-catenin. In the nucleus, down-regulation of Ku70 increases the physical interaction between β-catenin and TCF4, therefore inducing the transactivation of the downstream target genes [15]. Here we show that the cross-talk between DNA-PK and β-catenin in the centrosome is regulated by SF-1. Thus the function of β-catenin in different subcellular compartment is regulated by distinct mechanism.

### SF-1 controls the abundance of cyclin A in the centrosome

**Figure 7:**
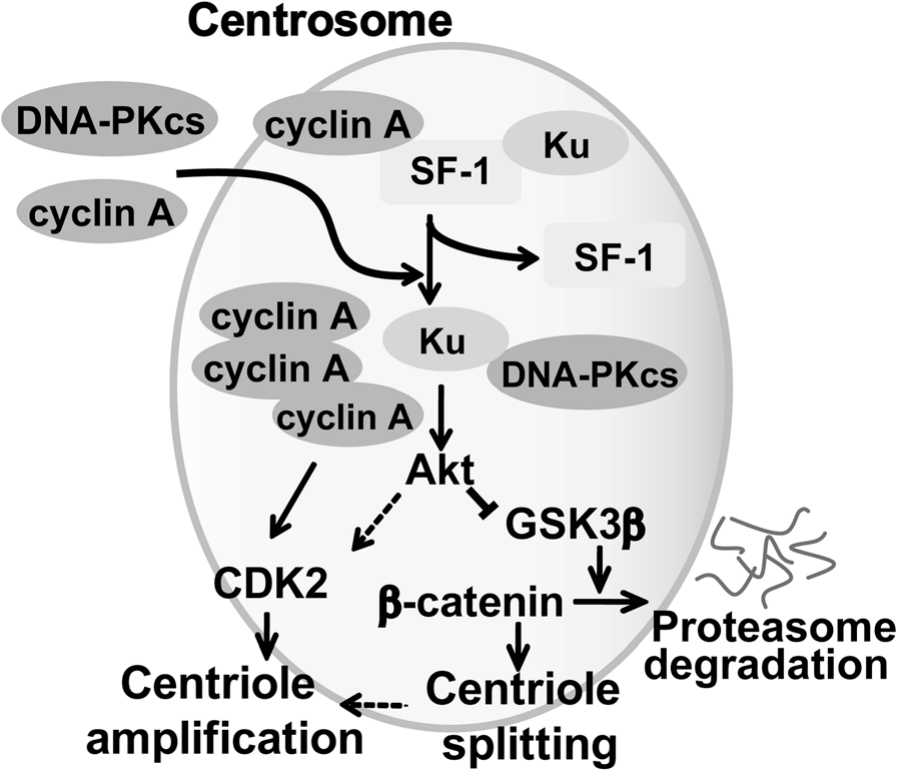
**Proposed model of SF-1 action in the centrosome.** In the centrosome, SF-1 interacts with Ku and cyclin A to prevent the DNA-PK/Akt signaling cascade. In the absence of SF-1, DNA-PKcs and cyclin A are recruited to the centrosome, leading to the activation of DNA-PK and Akt. This signaling cascade leads to GSK3β inactivation and β-catenin accumulation in the centrosome, therefore inducing centriole splitting. On the other hand, accumulation of cyclin A leads to CDK2 activation, thus facilitating centriole amplification. Solid arrows: proven activation step. Dotted arrows: indirect activation, perpendicular lines: inhibitory step.

We have previously shown that cyclin A participates in centrosome amplification caused by SF-1 depletion [10]. SF-1 interacted with cyclin A in the centrosome; this interaction appears to sequester cyclin A. In the absence of SF-1, cyclin A is recruited to the centrosome and centrosomal CDK2 activated [10]. CDK2 activity is required for centrosome duplication [16]. The recruitment of DNA-PKcs and cyclin A to the centrosome contributes to centrosome amplification in SF-1 deficient cells. Thus we propose that SF-1 controls centrosomal CDK2 activity by preventing aberrant accumulation of cyclin A in the centrosome (Figure 7).

We found that SF-1 interacted with cyclin A in the centrosome. SF-1 also interacts with Ku70, which has been identified as a binding partner of cyclin A in the centrosome [17]. Thus SF-1 may form a complex with Ku70 and cyclin A in the centrosome (see our model in Figure 7). This interaction appears to be independent of its DNA binding domain, as the SF-1-D70 mutant devoid of the DNA-binding domain still had normal function in the centrosome. We have shown that SF-1 depletion induces cyclin A accumulation and CDK2 activation in the centrosome [10]. Once SF-1 is depleted, the composition of the complex would be changed, more cyclin A would be recruited followed by activation of CDK2 in the centrosome. This will lead to centrosome amplification. Thus the role of SF-1 appears to depend on its interaction with Ku70 and cyclin A so as to sequester them. In the absence of SF-1, Ku70 will interact with and activate DNA-PKcs, while cyclin A will recruit and activate CDK2 in the centrosome. The increased activities of DNA-PK in the centrosome will lead to centriole splitting followed by centrosome amplification.

## Methods

### Cell culture and drug treatment

Mouse adrenocortical Y1 and testicular Leydig MA-10 cell lines were grown in Dulbecco’s modified Eagle medium (DMEM)-F12 medium supplemented with 10% fetal bovine serum at 37°C in a humidified atmosphere at 5% CO_2_. Human embryonic kidney 293FT cells were grown in Dulbecco’s modified Eagle medium (DMEM) medium supplemented with 10% fetal bovine serum at 37°C in a humidified atmosphere at 5% CO_2_. For drug treatment, cells were incubated with or without 1 mM vanillin 48 h before analysis. The performance of all experiments were approved by Academia Sinica biosafety committee.

### Protein depletion or over-expression

A lentiviral system for gene silencing was obtained from the National RNAi Core Facility (Institute of Molecular Biology, Academia Sinica, Taipei, Taiwan). Short hairpin RNA (shRNA)-encoding pLKO.1 vectors were as follows:

- pLKO.1-shluc (target sequence: 5′-cctaaggttaagtcgccctcg-3′)
- pLKO.1-shsf1#2 (target sequence: 5′-cgcaccatcaagtctgagtat-3′)
- pLKO.1-shsf1#3 (target sequence: 5′-cgtctgtctcaagttcctcat-3′)
- PLKO.1-shDNAPKcs#5 (target sequence: 5′-gctgcatacaactgtgccatt-3′)

A lentiviral system was also used for tetracycline-inducible protein expression. The *pTrip-aOn* plasmid was constructed by inserting the coding sequence of the Tet-On advanced transcriptional activator into *pTrip-IRES-neo* downstream from the elongation factor 1 alpha promoter. The plasmids for overexpression of EYFP, EYFP-D70-SF-1 and 3-FLAG-SF-1 were constructed by inserting the coding sequence of enhanced yellow fluorescent protein (EYFP), or wild-type or N terminal truncated (D70) SF-1 into *pAS4w.1.Pbsd*, which contains seven copies of modified *tetO* sequence.

293FT cells were cotransfected with packaging vectors *pCMVdelR8.9*1 and *pMD.G* as well as transfer vectors, which were either *pLKO.1*-derived plasmids encoding shRNA or *pAS4w.1.Pbsd*–based protein overexpression plasmids according to the protocols provided by the Taiwan National RNAi Core Facility. 16 hours after co-transfection, culture media were removed and fresh media were added and incubated for an additional 24 h. The medium were collected and new medium were added for further 24 h, then the new media were collected and pooled with previously collected one. The pooled viral harvests were purified by removing cell debris after centrifugation at 1250 rpm for 5 min to remove cell debris and stored at -80°C for future use.

For the generation of tetracycline-inducible SF-1 overexpression cells, Y1 cells were infected with *pTrip-aOn* lentivirus at a multiplicity of 3 and incubated for 24 h before G418 (500 μg/ml) selection. Infected cells were selected by G418 for more than one week until no further cell death was observed, indicating all the un-infected cells were removed. After selection, pooled G418-resistant cells were infected again by AS4w based, EYFP-, EYFP-SF1- or EYFP-SF1-D70-expression lentivirus at a multiplicity of 3 and incubated for 24 h before selection with 10 μg/ml blasticidin. The infected cells were selected again by blasticidin for more than one week until no further cell death was observed. The surviving cells were pooled, amplified, and treated with 1 μg/ml doxycycline for 48 h to induce transgene expression. To eliminate endogenous SF-1 expression, doxycycline and shRNA-encoding *shsf1#3* lentivirus were added to the cells simultaneously.

Y1 cells were transiently transfected with *pcDNA5-3-FLAG-SF-1* using Lipofectamine and Plus Reagents (Invitrogen, Carlsbad, CA) according to the manufacturer’s instructions. Five microgram *pcDNA5-3-FLAG-SF-1* [18] and 15 μl Plus Reagent were first mixed in 500 μl Opti-MEM medium (Life Technologies, Grand Island, NY) for 15 min, then mixed with pre-mixed 15 μl Lipofectamine in 500 μl Opti-MEM medium and incubated at room temperature for 20 min before the mixture was layered onto cells in 1 ml DMEM/F12 medium.

### Reporter assay

To measure SF-1 transcriptional activity, wild-type or D70 truncated SF-1 stable cells were co-transfected with a *CYP11A1*:*luciferase* reporter plasmid and *pRLuc* that encodes Renilla luciferase as an internal control. Luciferase activities were measured 24 h after transfection. The firefly luciferase activities were normalized with Renilla luciferase activities.

### Antibodies

The following antibodies were obtained commercially: anti-γ-tubulin, polyclonal anti-FLAG, anti-Cyclin A, monoclonal anti-FLAG M2, anti-α-tubulin and anti-acetylated-α-tubulin (all from Sigma, St. Louis, MO), anti-Cyclin E, anti-CDK2 phospho-Thr160, anti-Akt and anti-Akt phospho-Thr308 (Cell Signaling, Beverly, MA), anti-centrin 20H5 (Millipore, Billerica, MA), polyclonal anti-β-catenin and anti-GSK3β (Abcam, Cambridge, UK), anti-Ku70 (Genetex, Trvine, CA), anti-DNA-PKcs, and anti-DNA-PKcs phospho-Thr 2609 (Santa Cruz Biotech, Santa Cruz, CA). The immune sera against SF-1 have been described previously [19].

### Immunofluorescence microscopy

Cells were grown on glass coverslips at 37°C before fixation with ice-cold methanol at -20°C for 6 min. To visualize centriolar α-tubulin staining, cells were treated with 30 μM nocodazole on ice for 1 h to depolymerize microtubule networks, followed by brief extraction with saponin (20 ng/ml) for 2 min and fixation with ice-cold methanol for 5 min. After blocking with 5% BSA for 1 h, cells were incubated with antibodies for 24 h at 4°C, washed extensively with phosphate-buffered saline (PBS), and incubated with fluorescein isothiocyanate-conjugated and Cy3-conjugated secondary antibodies (Invitrogen, Carlsbad, CA) and 4′, 6-diamino-2-phenylindole (DAPI, 0.1 μg/ml) for 1 h in the dark. After extensive washing, the coverslips were mounted in 50% glycerol on glass slides. Fluorescent cells were examined with an AxioImager Z1 fluorescence microscope or an LSM 510 confocal microscope (both from Zeiss, Oberkochen, Germany). The numbers of centrosomes and centrioles from more than 100 cells were counted under the microscope in three independent experiments and shown as mean ± standard deviation. Student’s *t* test was performed to analyze the difference between different groups as indicated.

### Subcellular fractionation

Subcellular fraction and crude centrosomes were prepared by modifying a published procedure [20]. Briefly, 4 × 10^9^ cells were treated with nocodazole (10 μg/ml) and cytochalasin B (5 μg/ml) for 60 min, followed by sequential wash with cold PBS, 8% (w/w) sucrose in 0.1 × PBS, and 10 mM Tris–HCl, pH 8.0. Cells were then lysed in lysis buffer (10 mM Tris–HCl, pH 8.0, 0.5% NP-40 and 0.1% β-mercaptoethanol), and the cell lysate was centrifuged at 2,000 g. The nuclear pellet was further lysed with lysis buffer containing 0.5% NP-40, 300 mM NaCl, 1 mM EDTA, and the protease inhibitor cocktail (Roche, Mannhein, Germany).

The cytoplasmic fraction was centrifuged again at 10,000 g for 1 h on a 50% sucrose (w/w) cushion layer. Supernatant was removed and the resulting sucrose cushion containing the concentrated centrosome was centrifuged over a discontinuous 75%, 50% and 40% (w/w) sucrose gradient at 35,000 g. Samples (300 μl/fraction) were collected from the bottom of the tube and the crude centrosomes were enriched in sucrose fraction #5 at about 55-60% density.

### Immunoprecipitation assay

Protein extracts were incubated with specific antibodies for 1 h at 4°C before further incubation with protein-G beads for 1 h at 4°C. The beads were washed with buffer containing 10 mM Tris–HCl, pH 8.0, 120 mM NaCl, 1 mM EDTA, 1 mM PMSF, and the protease and phosphatase inhibitor cocktail. The samples were eluted with 3-FLAG peptide or sample buffer.

### Quantification of immunblots

The images of Western blot analysis on the X-ray film were scanned and saved as the Tagged Image File (TIF) format. The intensity of each protein band was quantified by the Image J software (NIH, Bethesda, MD). The intensities of protein bands were normalized against those of the internal controls and shown as relative units.

## Abbreviations

SF-1: Steroidogenic factor 1
DNA-PK: DNA-dependent protein kinase
DNA-PKcs: Catalytic subunit of DNA-PK
PCM: Pericentriolar material
D70-SF-1: SF-1 that lacks the N-terminal DNA-binding domain
Nek2: NIMA-related protein kinase 2
DMEM: Dulbecco’s modified Eagle medium
DAPI: 4′, 6-diamino-2-phenylindole
EYFP: Enhanced yellow fluorescent protein
